# *Rhizobium leguminosarum* bv. *viciae* 3841 Adapts to 2,4-Dichlorophenoxyacetic Acid with “Auxin-Like” Morphological Changes, Cell Envelope Remodeling and Upregulation of Central Metabolic Pathways

**DOI:** 10.1371/journal.pone.0123813

**Published:** 2015-04-28

**Authors:** Supriya V. Bhat, Sean C. Booth, Seamus G. K. McGrath, Tanya E. S. Dahms

**Affiliations:** 1 Department of Chemistry and Biochemistry, University of Regina, 3737 Wascana Parkway, Regina, SK, S4S 0A2 Canada; 2 Department of Biological Sciences, University of Calgary, 2500 University Dr, NW Calgary, AB, T2N 1N4 Canada; University of Illinois at Urbana-Champaign, UNITED STATES

## Abstract

There is a growing need to characterize the effects of environmental stressors at the molecular level on model organisms with the ever increasing number and variety of anthropogenic chemical pollutants. The herbicide 2,4-dichlorophenoxyacetic acid (2,4-D), as one of the most widely applied pesticides in the world, is one such example. This herbicide is known to have non-targeted undesirable effects on humans, animals and soil microbes, but specific molecular targets at sublethal levels are unknown. In this study, we have used *Rhizobium leguminosarum* bv. *viciae* 3841 (*Rlv*) as a nitrogen fixing, beneficial model soil organism to characterize the effects of 2,4-D. Using metabolomics and advanced microscopy we determined specific target pathways in the *Rlv* metabolic network and consequent changes to its phenotype, surface ultrastructure, and physical properties during sublethal 2,4-D exposure. Auxin and 2,4-D, its structural analogue, showed common morphological changes *in vitro *which were similar to bacteroids isolated from plant nodules, implying that these changes are related to bacteroid differentiation required for nitrogen fixation. *Rlv* showed remarkable adaptation capabilities in response to the herbicide, with changes to integral pathways of cellular metabolism and the potential to assimilate 2,4-D with consequent changes to its physical and structural properties. This study identifies biomarkers of 2,4-D in *Rlv* and offers valuable insights into the mode-of-action of 2,4-D in soil bacteria.

## Introduction

Environmental disturbance both through anthropogenic and natural sources is a global issue, transforming habitats and creating stress for a wide variety of organisms. It is increasingly recognised that there is a need to assess the effects of chemical pollutants at the molecular level to better understand their impact on the environment [1]. The herbicide 2,4-dichlorophenoxyacetic acid (2,4-D), as one of the most widely applied herbicides in the world, is one such example of an anthropogenic chemical pollutant. As a structural analogue of a naturally occurring plant hormone which induces cell growth, elongation and division, indole acetic acid (IAA, auxin), 2,4-D mainly targets dicotyledonous weeds. Unlike auxin, 2,4-D is stable and resistant to plant auxin degradation pathways and so accumulates in plant cells causing oxidative-induced damage, loss of membrane integrity, senescence, foliar damage, accumulation of abscisic acid and ethylene, and eventually plant death in sensitive dicots [2,3]. This herbicide is known to cause carcinogenic free radical reactions, mutagenicity, birth defects, tissue defects, DNA damage and apoptosis and a wide range of other negative impacts in mammals, fishes, birds and humans [4]. However, the impact of 2,4-D at the molecular level in soil bacteria is only beginning to be understood [5]. In agriculture, amine salts, alkali or esters of 2,4-D are applied at 0.2–2 kg ha^-1^ of active ingredient, while granular herbicides are used as aquatic herbicides at 1–122 kg ha^-1^ [5]. While environmentally relevant levels of 2,4-D have been determined to be 5 mg kg^-1^ soil [6], bacterial exposure depends on several environmental factors such as moisture, organic content and the presence of 2,4-D assimilating organisms [5,7], such that soil bacteria are exposed to a range of sublethal concentrations.

*Rhizobium leguminosarum* bv. *viciae* 3841 (*Rlv*), a beneficial soil bacterium belonging to the family of α-proteobacteria, fixes atmospheric nitrogen by forming a symbiotic relationship with leguminous roots. Organisms of this genus are crucial for soil fertility as they are capable of infecting plant roots and forming nodules within which they convert atmospheric nitrogen to ammonia available for plant uptake [8]. This key symbiotic relationship can be affected by a number of factors, including abiotic chemical stresses such as fertilizers and pesticides [9], making the study of their impact on rhizobial physiology extremely important. Auxin is produced by free living rhizobia and is thought to play a role in nodule development based on its increased transport to and accumulation at the site of nodule formation [10]. Auxin and 2,4-D are both known to induce nodular outgrowths, also called para-nodules on monocot roots, which in *Azospirillum brasilense* is accompanied by increased colonization and a general increase in nitrogenase activity post treatment [11–13]. Evidence regarding the effects of 2,4-D on rhizobial nodulation is limited and controversial [14,15] and does not address the role of plant- and rhizobia-derived auxin on rhizobial physiology.

Rhizobia are model soil bacteria capable of persisting for years in the soil environment between symbiotic phases. Rhizobia are known to produce excess amounts of surface and exopolysaccharides as one of their main adaptation mechanisms to desiccation and other abiotic stresses [16]. *Rlv* 3841 is a spontaneous streptomycin resistant mutant of a soil isolate for which the complete genome sequence became available during the past decade [17]. While this strain is possibly one of the most well studied of its genera, very few studies have examined its stress signaling pathways [18,19].

Studies exploring the effects of 2,4-D on soil bacteria indicate that it is capable of causing toxic responses in *Escherichia coli* [20,21], *Corynebacterium glutamicum* [22], *Deftia acidovorans* [23], *Burkholderia sp*. [24], *Saccharomyces cereviciae* [25–27], *Pseudomonas putida* [28] and *Azospirillum brasilense* [13,29–31], but the molecular basis of its mechanism is not fully understood. In rhizobial species 2,4-D affects growth, protein content and membrane fluidity [32–34]. This study identifies the specific cellular pathways targeted by 2,4-D and the consequent ultrastructural and phenotypic changes to *Rlv*.

Metabolomics, a method that unbiasedly determines differences in metabolite profiles between treated and control sample sets, combined with atomic force microscopy (AFM) which probes cell ultrastructure and mechanical properties constitutes a novel approach for characterizing toxicological effects. Metabolomics detects the specific pathways altered by any given stimulus, establishing metabolic fingerprints of external stress factors [35], while atomic force microscopy (AFM) can determine cell structure from mm to nanometer resolution and physical properties down to the pico-newton scale [36]. AFM has been widely used to characterize surface ultrastructural changes, mechanical properties and single molecule mapping in biological systems [37–41].

In the present study, *Rlv* 3841 was exposed to sublethal levels of 2,4-D while tracking surface ultrastructure, physical properties and metabolism. This is first study of its kind in this bacterium, providing useful insights into the mode-of-action of this herbicide and specific *Rlv* stress response pathways that may serve as biomarkers of 2,4-D exposure. *Rlv* treated with 2,4-D exhibit remarkable adaptation, alteration of vital metabolic pathways and consequent changes to the cell envelope and phenotype. 2,4-D-induced morphological changes were similar to those effected by auxin *in vitro* and bacteroids directly isolated from pea root nodules, indicating that 2,4-D likely induces auxin-like non-target response in *Rlv*.

## Materials and Methods

The 2,4-D amine formulation (w/w % 84.21 2,4-D, 0.5 Triton-X-100, 1.5 EDTA, 1.41 of 60% dimethylamine aq. solution, and 12.38 of soft water; analysis by Interprovincial Cooperative Limited (Agri Products Department, Winnipeg, Canada)) was purchased from Viterra, Canada. All other chemicals were analytical grade and purchased from Sigma-Aldrich unless otherwise noted. Ultrapure deionized water (18 MΩ, Barnstead Nanopure, Thermo Scientific) was used for media and sample preparation.

### *Rlv* growth conditions and MIC assays

*Rhizobium leguminosarum* bv. *viciae 3841 (Rlv)* was maintained on Ca^2+^ rich tryptone-yeast-streptomycin (TY-st) media [42] (5 g/L Tryptone, 3 g/L yeast extract and 0.5 g/L CaCl_2_•2H_2_O) at 30°C. A 24 h culture was used as a stock for all inoculations.

The MIC (minimum inhibitory concentration) of the 2,4-D formulation was determined by growing *Rlv* for 24–48 h at 30°C in TY-st media tubes containing increasing concentrations (0–1 mM) of filter sterilized 2,4-D formulation. A formula control, consisting of all formulation ingredients except 2,4-D, and sample controls containing deionized water in place of formulation, were tested in parallel.

### Microscopy

Standard sized coverslips (22 mm × 22 mm) were cleaned and coated with poly-L-Lysine [38]. Approximately 500 μL of the 2,4-D-treated (0.021–0.42 mM) and control 24 h broth cultures (OD ~ 1) were pipetted onto poly-L-Lys-coated coverslips, incubated (30 min), rinsed with water and stained (crystal violet 0.1 mg mL^-1^) for light microscopy (LM).

To study dose-response relationships at higher resolution, suitable sublethal concentrations were chosen based on the morphological effects observed by LM (Olympus BX51). Samples for scanning electron microscopy (SEM) were prepared as for LM, but without staining. Coverslips incubated with culture were rinsed with VMM buffer (0.1 M; pH 7) and fixed (3.7% formaldehyde, 0.2% Triton-X-100 in phosphate buffer pH 7) for 10 min. The coverslips were rinsed with sterile water and air dried. Samples were substituted by immersion in increasing concentrations of ethyl alcohol (20–100% v/v in water) for 10 min after fixation. The coverslips were mounted onto specimen stubs and copper strips were attached to increase conductivity. Samples were sputter coated with gold (SC7620 mini sputter coater, Emitech) for 120 s and imaged by SEM (JEOL JSM-6360) at 8 keV using the secondary electron imaging mode.

For ultra-high resolution images to study the effects of 2,4-D on the cell wall surface ultrastructure, chemical characteristics, and phenotype, samples were imaged by AFM at suitable sublethal concentrations. Sample preparation for AFM was similar to that for SEM but without drying and coating. Following fixation, coverslips were dried overnight and imaged by AFM (minimum of 3 different samples, 2 areas on each sample, 10 bacteria each). Similar sample preparation protocols were followed for sample treated with IAA (0.4, 0.9 mM) and benzoic acid (0.4, 1 mM). Topography, force spectroscopy and quantitative imaging data was collected using a NanoWizard 3 AFM (JPK, Germany) equipped with soft contact mode silicon nitride (Si_3_N_4_) cantilevers (HYDRA2R-50NG-10, AppNano) having a calibrated spring constant [43] (k) of 0.092±0.03 Nm^-1^ and a nominal tip radius of <10 nm. Topography images served as a template for force curves, collected using a constant approach velocity (0.1 μm s^-1^) in triplicate at three spots on the center axis of 30 bacterial cells. Quantitative imaging (QI mode, JPK, Germany) was used to collect adhesion maps of treated and control samples.

### Image Processing and Analysis

AFM images were leveled, adjusted for optimal contrast, and the shadow effect used only for visual clarity in figures. Surface roughness was calculated from ten different bacterial surfaces for at least three different samples based on the following equation,

$${\text{R}}_{\text{a}}=\frac{1}{n}\overset{n}{\underset{i=0}{\sum }}\left|Z_{i}-\bar{Z}\right|$$ (1)

where, *Z_i_* is the average height of surface features and *Z_i_* is the height of each surface feature.

Cantilever deflection was converted to force (JPK image processing software) using the cantilever spring constant. Surface adhesion values were calculated from the e-f segment on the retraction force curve (see results) and plotted using GraphPad Prism (GraphPad 5.01, La Jolla, CA, USA).

Young’s modulus was calculated using the Hertz model (JPK software) [44] for a tetrahedral tip,

$$F=\frac{E}{1-v^{2}}\;\frac{\tan\alpha }{\surd 2}\delta^{2}\;\alpha =\frac{\tan\alpha }{\surd 2}\delta \;\;\;\;$$ (2)

where, E is the Young’s modulus, *δ* is the indentation, *v* is Poisson’s ratio (0.5 for biological samples) and *α* is the face angle of the cantilever.

Standard deviation was used to determine variations in sample sets. A paired student’s t-test (GraphPad, 5.01) was used to assess differences between treated samples and controls.

### Plant assays

*Rlv* bacteroids were isolated from pea root nodules and imaged by AFM for phenotypic and ultrastructural comparison as described in the supplementary information (S1 Methods).

### Metabolite extraction and analysis

Six replicates of *Rlv* treated with formula control, control and 0.4 mM 2,4-D were grown overnight in TY broth. Cells were quenched and extracted according to Booth et al. [45]. Aqueous extracts were dried (SpeedVac, (Savant-Thermoquest, DDA)) and stored at -80°C. Samples were thawed, treated with methoxylamine hydrochloride (50 μL; 20 mg mL^-1^), incubated (2 h; 37°C, 25 rpm), silylized [45] using N-methyl-N-trimethylsilyltrifluoroacetamide (50 μL MSTFA), incubated on a shaker (45 min, 37°C, 25 rpm), diluted (400 μL hexane), centrifuged (7 min, 14,000 × g) and 200 μL transferred to a gastight vial. Sample and alkane standards (1 μL) were injected (splitless injection; 275°C) onto a GC-MS (Waters GCT premier MS) DB5-MS column (30 m-0.25 mm i.d. × 0.25 μm) for separation (80°C for 1 min, increased by 12°C min^-1^ to 320°C, held 8 min) and analysis (range = 50–800 m z^-1^), using helium as the carrier gas (1.2 mL min^-1^).

### Spectral processing and multivariate statistical analysis

GC-MS spectra were converted to net-CDF files, peaks detected and compounds identified (Metabolite detector software tool [46]). Retention indices were calibrated using alkane standards (C10-C30), and identified metabolites were batch quantified and exported. Metabolites found in at least 60% of the replicates were subsequently analyzed, with remaining missing values imputed by k-means nearest neighbor (KNN) [47]. Data were normalized by median fold change, centered and unit-variance scaled for multivariate statistical analysis (Simca-P 12, Umetrics). General clustering trends and metabolite differences were assessed with unsupervised principal component analysis (PCA). Metabolite variations and good models (*R* ^*2*^ *Y* and *Q* ^*2*^ ~ 1) were confirmed with supervised orthogonal partial least square discriminant analysis (OPLS-DA) [48]. Reliability and significance (p < 0.05) of the OPLS models were tested with seven-fold Cross Validation Analysis Of Variance (CV-ANOVA). Shared and unique structures plots (SUS-plots) of variable influence on projection (VIP) and correlation coefficient (p(corr)) values were generated (GraphPad Prism), and metabolic pathways most representative of detected metabolites determined (MBrole pathway enrichment analysis) [49].

### ROS assay

Intracellular ROS was measured using the ROS-sensitive probe 2,7-dichlorodihydrofluorescein diacetate (DCFDA) according to Perez et al [50] for *Rlv* treated with 0–0.4 mM 2,4-D after 24 h incubation. The fluorescence intensity, directly indicating ROS levels, was measured using a BioTek microplate reader (Winooski, VT, US; λ _ex_ = 485 nm; λ _em_ = 528 nm).

### Protein carbonylation (PC) assay

The carbonylated proteins were detected using the dinitrophenyl hydrazine (DNPH) assay according to Semchyshyn et al [51]. *Rlv* cells treated with 0–0.4 mM 2,4-D were grown for 24 h and the nucleic acid free cell extracts were treated with four volumes of 4,4’-dinitrophenylhydrazine (dissolved in 2 M HCl) and incubated at room temperature for 1 h with vortexing every 25 min. Protein was precipitated using 20% TCA and pelleted by centrifugation (12,000 × g, 10 min). The pellet was washed three times using 1:1, ethyl acetate:ethanol to remove unreacted dinitrophenylhydrazine and then dissolved in 450 μL of 50 mM dithiothreitol in 6 M guanidine HCl. Carbonyl content was determined spectrophotometrically at 370 nm.

### MS and ICL enzyme assays

Malate synthase was assayed according to Ramirez-Trujillo et al [52] with minor changes. Briefly, *Rlv* was grown in 50 mL cultures in the presence of 0–0.4 mM 2,4-D until an O.D of ~1 was reached. Cells were harvested by centrifugation (12,000 × g), the pellet washed with 100 mM Tris HCl and suspended in the same buffer containing protease inhibitors (protease inhibitor cocktail tablets, Roche) and sonicated on ice for 2 min in 30 s/1 min on/off cycles. Extracts were isolated by centrifugation (20,000 × g, 10 min) and MS activity determined by the glyoxylate-dependent release of free CoA from acetyl-CoA. The assay mixtures (0.2 mL, 96 well plate) contained 100 mM Tris HCl (pH 7.5), 10 mM MgCl_2_, 2.5 mM glyoxylic acid, and *Rlv* extract. The reaction was initiated by addition of acetyl-CoA to a final concentration of 0.43 mM, incubated (room temperature, 10 min), terminated, color developed by the addition of 5,5’-dithiobis(2-nitrobenzoic acid) (Life technologies) and its absorbance measured (412 nm) using a microplate reader (BioTek). Negative controls did not contain glyoxylate.

ICL activity was measured as previously described [52]. The reaction buffer (pH 6.8) containing 50 mM MOPS, 5 mM MgCl_2_, 5 mM cysteine HCl, 1 mM EDTA and 4 mM phenyldrazine HCl was added to the *Rlv* extract, and isocitric acid added to a final concentration of 12.5 mM to initiate the reaction. The increase in the level of the phenylhydrazone derivative of glyoxylate was measured as absorbance at 324 nm. Negative controls lacked isocitrate.

Absorbance values for the PC, MS and ICL assay were normalized with protein concentrations determined by the Bradford method, and were repeated a minimum of three times for statistical validity.

## Results

### Sublethal levels of 2,4-D alters phenotype, surface ultrastructure and elasticity

**Fig 1:**
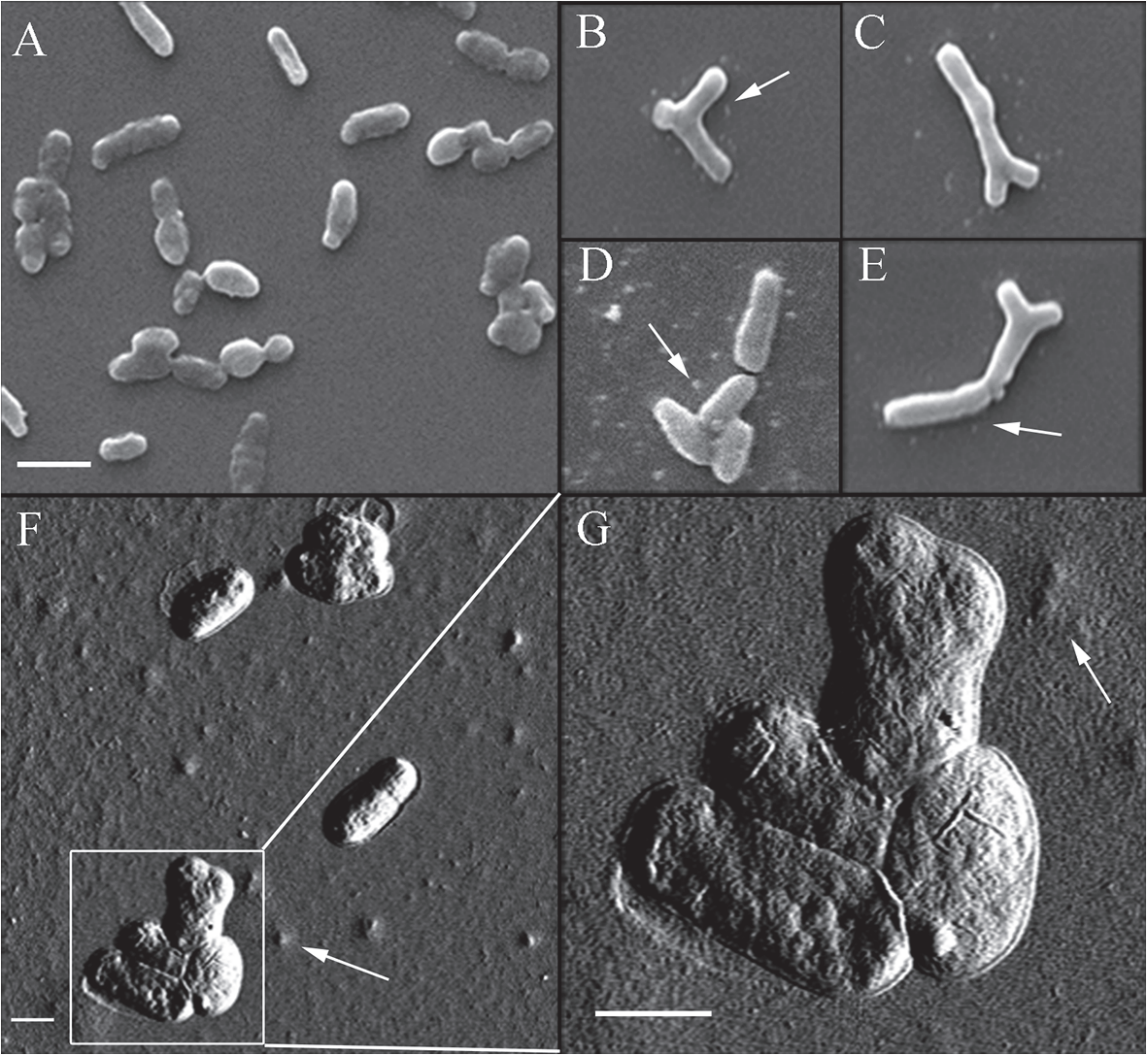
SEM and AFM images highlight the infrequent phenotype in 2,4-D treated *Rlv*. Most of the untreated (A) and treated (D) *Rlv* appeared normal but a small number of the 2,4-D (0.4 mM) treated cells appear ‘Y’ shaped (C, E, F, G) and some appeared to branch (B). F and G show one (top) of the four clustered cells with clear demarcation for initiation of the ‘Y’ shaped phenotype. Arrows indicate extracellular matrix on the substrate surface. Scale bar A-E 2 μm, F and G 1 μm.

*Rlv* exposed to 2,4-D had a MIC of 0.6 mM and were able to grow at 0.4 mM 2,4-D with sufficient cell density to be used as the highest sublethal concentration for microscopy, enzyme assays and metabolomics studies. LM did not show observable morphological differences between the control and treated *Rlv*, however SEM images of *Rlv* cells exposed to 0.4 mM 2,4-D exhibited a Y-shaped phenotype (Fig 1C, 1E, 1F and 1G) some of which appeared to branch (Fig 1B) at a frequency of 32 ± 11% compared to the formula (4.6 ± 1.1%) and control (3.8 ± 0.8%) samples (p <0.0001, n≥200) (S3 Fig). These cells were much larger than the untreated rod-shaped phenotype.

**Fig 2:**
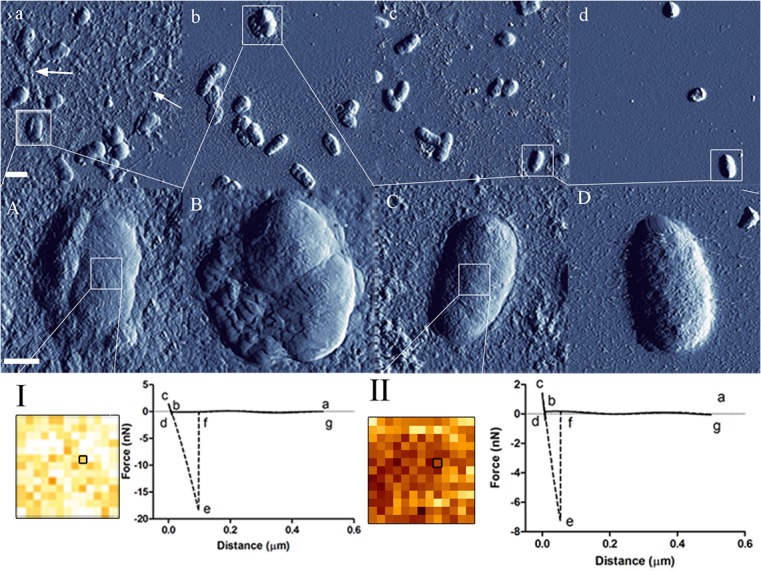
AFM and QI images with force curves illustrate cell surface remodelling with 2,4-D exposure. *Rlv* treated with 0.4 mM (a, A), 0.2 mM (b, B) 2,4-D have markedly altered surfaces compared to those of formula (c, C) and sample controls (d, D). Arrows indicate extracellular matrix on the substrate surface, only observed for *Rlv* treated with 2,4-D. Images a-d are low resolution (300 × 300) and A-D are high resolution (500 × 500). Scale bars, a-d = 1 μm, A-D = 0.5 μm. I and II show quantitative imaging data and representative force curves corresponding to one pixel at the center of the cell surface adhesion map for treated and control samples, respectively. From the force curves (a-c extend, c-g retract) the e-f segment is used to measure the adhesion force and b-c used to calculate Young’s moduli. QI adhesion maps indicated that treated cells had a much higher (lighter pixels) tip adhesion than the control (darker pixels).

AFM indicated that *Rlv* treated with 0.4 mM 2,4-D had a two fold increase in surface roughness (12.12 ± 4.29 nm) compared to the formula (6.52 ± 1.5 nm) and sample controls (6.65 ±1.5 nm) (n = 30, p<0.0001). At 0.4 mM, SEM of 2,4-D-treated *Rlv* exhibited extracellular material around the cells, which were clearly apparent in the high resolution AFM images. The e-f segment of the force retraction curve collected by force spectroscopy and quantitative imaging indicates adhesion between the bacterial surface and the negatively charged AFM tip, while the b-c segment of the approach curve was used to calculate Young's moduli representing envelope compliance (Fig 2I and 2II). Cells treated with 0.4 mM 2,4-D were significantly more (n ≥ 60, p < 0.0001) adhesive (15.9 ± 8 nN) compared to the formula (10 ± 3 nN) and the sample control (8.25 ± 3.16), and had a significantly higher (n ≥ 60, p < 0.0001) Young’s modulus (87 ± 24 MPa) compared to the formula (64 ± 9 MPa) and sample control (61 ± 16 MPa).

### Auxin plays a role in rhizobial differentiation *in vitro*

Since 2,4-D is a structural analogue of IAA and has been presumed to play a role in rhizobial differentiation, *Rlv* were treated with 0.4 and 0.9 mM IAA. *Rlv* showed a unique phenotype at all concentrations, with some cells appearing to branch and others producing bud-like extensions with a clear demarcation from which the daughter cell appeared to separate (S1A Fig, S1B Fig and S2A, S2B Fig). These differentiated cells appeared at a frequency of 77 ± 15% for the 0.4 mM IAA and 79 ± 10% for the 0.9 mM IAA treated cells compared to the formula (4.6 ± 1.1%) and control (3.8 ± 0.8%) samples (p < 0.0001, n ≥ 200) (S3 Fig). Benzoate treated samples, used as a negative control, had frequencies at 1 mM (3.8 ± 0.9%) and 0.4 mM (2.6 ± 0.2%) comparable to the control and formula samples (S3 Fig). *Rlv* treated with 2,4-D and IAA had phenotypes similar to differentiated bacteroids isolated from legume root nodules (S2 Fig c, C).

### Quantitative metabolomic analysis of *Rlv* under 2,4-D stress

**Fig 3:**
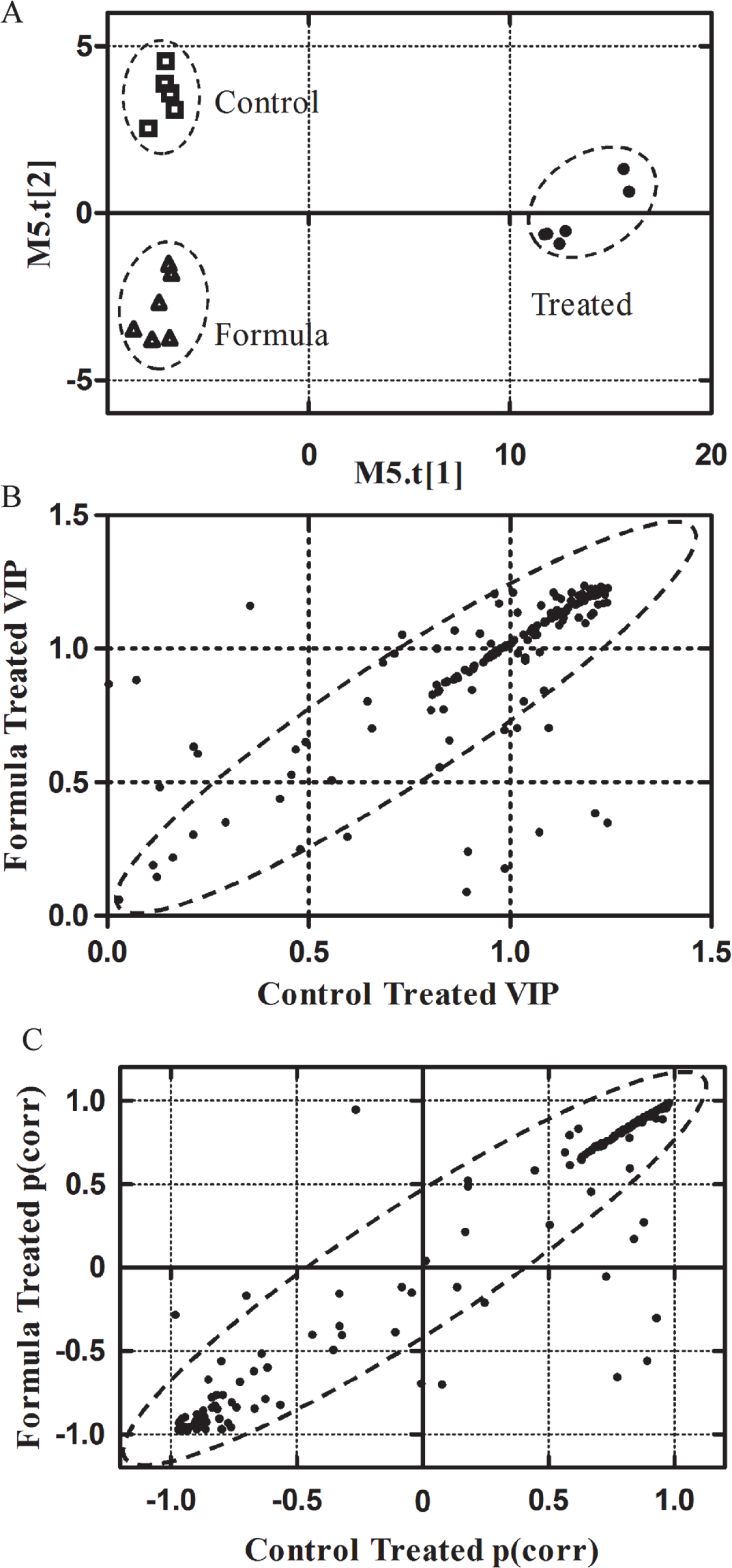
OPLS-DA (A) scores plot and SUS plots (B, C) for the multivariate statistical analysis. The OPLS-DA (orthogonal partial least square discriminant analysis) scores plot (A) shows clustering of the metabolite profiles for control, formula and treated samples, indicating good models with strong separation. SUS (Shared and Unique Structures) plots constructed for VIP (variable influence on projection) (B) and p(corr) values (C) show significant metabolites and metabolite correlation, respectively, with shared features. VIP and p(corr) values from formula and control samples were compared with those from treated samples. The diagonally aligned metabolites (highlighted with an ellipse) in both the plots show the similarity between the control and formula OPLS profiles indicating that the metabolic effects are mainly due to 2,4-D rather than other formulation components.

Optimized analytical conditions and statistical models achieved a good separation between the treated and control metabolic profiles. PCA analysis showed clustering of the treated and the two controls, indicating good separation between the control and treated groups. OPLS-DA supervised pairwise models had strong *R2Y* and *Q2* values, indicative of good cross-validation (Table 1) [53], with significantly different metabolite profiles for the treated samples compared to the two controls (Fig 3 A). A total of 175 metabolites were detected in all replicate sample sets out of which only 87 were used for further statistical analysis, comparable to similar approaches [54]. A total of 60 metabolites having a VIP score of >1, indicating above average influence on the model, were chosen for downstream pathway analysis. SUS (shared and unique structures plot) were created to determine the common general trend in the significant and non-significant metabolites. A VIP > 1 indicates metabolites that are significantly altered with 2,4-D treatment. VIP SUS plots (Fig 3 B) show the formula- and control-treated models to have similar trends with significantly altered metabolites in the 2,4-D-treated model, indicating that changes in the metabolome were mainly attributable to 2,4-D exposure. The correlation coefficient, p(corr), is positive for metabolite levels that increased or negative for those that were reduced in response to 2,4-D treatment. P(corr) SUS plots also showed a similar type of correlation between the control-treated and formula-treated models, with very few metabolites showing a change unique to the formula ingredients (Fig 3 C).

**Table 1:** OPLS-DA model statistics.

Model	*R2Y* ^*†*^	*Q2* ^*†*^	CV-ANOVA
All class	0.446	0.358	0.0005
Control-treated	0.982	0.976	3.05E-07
Formula-treated	0.985	0.980	2.30E-08
Control-formula	0.864	0.464	0.367*

*High CV-ANOVA value for control-formula model indicates lack of a good OPLS-DA model for the separation of control and formula sample sets due to the lack of statistically significant difference.

^*†*^ *R2Y* and *Q2* values close to 1 for control-treated and formula treated models indicate significant differences in the metabolite profiles between the sample sets. Absence of statistically significant differences between control and formula samples is shown by low *R2Y* and *Q2* values for the all class and control-formula models. These results indicate that changes in the metabolic profiles are mainly due to 2,4-D rather than the formula ingredients in the formulation.

### Secondary analysis shows major pathways are altered during adaptation to 2,4-D stress

**Fig 4:**
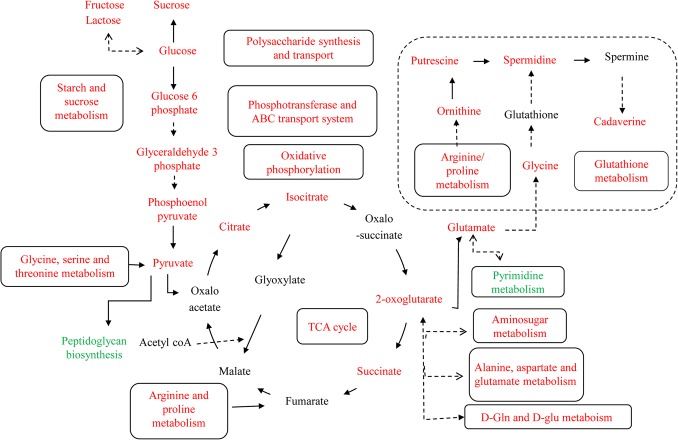
Metabolic pathways affected during adaptation to 2,4-D-induced stress. Metabolites and pathways colored in red and green are higher and lower, respectively in 2,4-D stressed *Rlv* compared with formula and sample controls. Metabolites colored in black were undetected. Enrichment analysis indicated that the majority of the metabolites identified were positively correlated with 2,4-D exposure, belonging to glycolysis, the TCA cycle, oxidative phosphorylation, ABC transport, the two component system and glutathione metabolism. Several pathways associated with pyrimidine and peptidoglycan metabolism were negatively correlated with 2,4-D exposure.

All 60 significant metabolites were assigned a unique KEGG (Kyoto Encyclopedia of Genes and Genomes) ID and we used *Rlv* libraries in MBrole [49] to study the affected pathway networks and biological interactions resulting from 2,4-D exposure. Only pathways containing metabolites with a VIP value > 1 and a p-value of < 0.05 (as determined by MBrole) were considered to represent effects attributable to 2,4-D exposure. Out of the 60 positively and negatively correlated metabolites, the majority were positively correlated with 2,4-D stress, which included amino acids (i.e. Pro, Glu, Thr, Asp, Met, Asn, Ala, Lys, Ser, Arg) and metabolites of the tricarboxylic acid (TCA) cycle, oxidative phosphorylation, ABC transport, the glyoxylate pathway and inositol phosphate metabolism (Fig 4). Fewer metabolites were negatively correlated, for which the majority belonged to pyrimidine metabolism and peptidoglycan biosynthesis (S1 Table). The total number of metabolites altered represented approximately 66% of all *Rlv* metabolic pathways (MBRole).

### 2,4-D causes ROS accumulation and protein carbonylation

**Fig 5:**
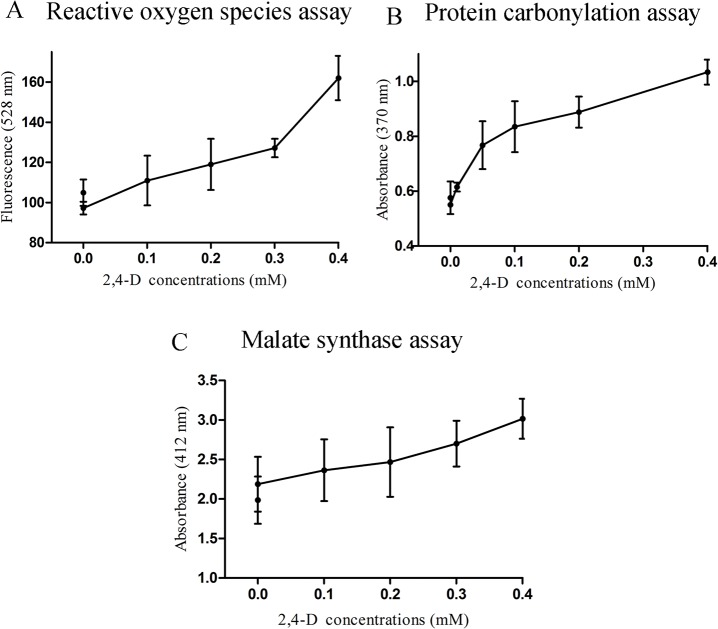
Assays of oxidative stress and glyoxylate metabolism with 2,4-D exposure show *Rlv* stress and adaptation. Plots show accumulation of reactive oxygen species (A), protein carbonylation (B) and malate synthase activity (C) as a function of 2,4-D exposure measured as ROS-sensitive DCFDA fluorescence intensity (528 nm), absorbance of dinitrophenyl hydrazone (370 nm) and absorbance of DTNB (412 nm), respectively. Absorbance values for the MS and PC assays were normalized with protein concentrations determined by Bradford assay. Data represent a minimum of three trials (p ≤ 0.05).

ROS, a direct indicator of oxidative stress, measured using the fluorescent probe DCFDA, showed a concentration dependent increase in oxidative stress (Fig 5 A) with 2,4-D exposure (p < 0.005). Protein carbonylation, one possible consequence of oxidative stress, was higher in 2,4-D treated *Rlv* cell extracts (Fig 5 B) and exhibited a concentration dependent increase in absorbance values (p < 0.0001).

### 2,4-D induces malate synthase activity

An increase in the metabolites associated with the glyoxylate pathway was rather intriguing, and therefore enzyme assays were used to assess this effect. Malate synthase activity was higher in *Rlv* exposed to 2,4-D compared to control samples (p < 0.05) and was concentration dependent (Fig 5 C), indicative of a direct effect of 2,4-D on the enzyme. Interestingly, there was no statistically significant difference in the isocitrate lyase activity between treated and control samples (S4 Fig).

## Discussion

*Rhizobium* is a beneficial soil bacterium having both a symbiotic and free living life style which requires a complex genome capable of altering physiology for both the heterogeneous soil environment and the more predictable life style in plant nodules [17]. Consequently, rhizobia have a robust and versatile metabolism, well suited to surviving external stress factors such as environmental pollutants. Herbicide exposed *Rlv* demonstrated a remarkable ability to adapt to 2,4-D-induced stress, with changes to phenotype, surface ultrastructure, physical properties and changes to integral pathways of cellular metabolism. As a structural analogue of auxin, it was not surprising that 2,4-D induced an auxin-like response in rhizobia *in vitro*, including a differentiation-like phenotype. This study provides valuable insights into 2,4-D sublethal toxicity and the associated adaptation of metabolic networks.

### 2,4-D and IAA alter the morphology of *Rlv in vitro*

SEM indicated that a small number of 2,4-D treated *Rlv* cells were abnormally shaped, some appearing to branch whereas others appeared similar to the ‘Y’-shaped bacteroids (Fig 1C, 1E, 1F and 1G) normally found only in root nodules. This observation is interesting but difficult to explain since this phenotype is normally associated with symbiosis during nitrogen fixation. Interestingly 2,4-D is a structural analogue of auxin, the plant hormone which is known to play a role during nodule development [55]. There is a strong overlap of proteomic changes in *Medicago trunculata* during early nodulation and within roots treated with auxin (van Noorden et al., 2007), and auxin synthesised by rhizobia promotes nodulation and host root growth in plants bearing indeterminate nodules [10]. Further, *Rlv* in which indole acetamide biosynthetic pathway proteins had been introduced and expressed produced root nodules in *Vicia hirsuta* containing up to 60-fold more auxin and having a higher nitrogen fixing capacity than those of the wild-type strain [56]. A *Azospirillum ipdC* mutant, producing lower amounts of auxin than the wild-type strain, had reduced nodulation and nitrogen fixation, demonstrating the role of bacterial auxin production in nitrogen fixation [57]. However, whether auxin plays a role in bacteroid differentiation, which is crucial for nitrogen fixation, remains unclear. To explore this idea and whether the 2,4-D-induced phenotype was a result of an auxin-like response, *Rlv* were exposed to a range of auxin concentrations. Indeed auxin induced morphological changes, with some cells appearing ‘Y-shaped’ like bacteroids and others appearing to branch and bud (S1A and S1B Fig and S2a, A Fig, S2b, B Fig). The frequency of non-rod-shaped cells was significantly higher (p < 0.0001) with auxin treatment compared to 2,4-D (S3 Fig), whereas benzoic acid was not capable of inducing such phenotypic changes (S1C Fig and S2d, D Fig). Bacteroids isolated from pea root nodules showed a phenotype (S2c, C Fig) similar to that of the auxin and 2,4-D treated cells, further evidence that this herbicide likely mimics auxin by activating *Rlv* signaling *in vitro* related to differentiation. If terminally differentiated, these rhizobia would not be available for nodule formation in legumes.

### 2,4-D alters the surface ultrastructure and physical properties of *Rlv*

*Rlv* exposed to a range of 2,4-D levels showed a dose-dependent change in surface features such as roughness, elasticity and adhesion (Fig 2). Changes in surface ultrastructure indicate cell wall macromolecular remodeling, which could be consistent with increased amino acids in the metabolite pool during 2,4-D exposure. *Acinetobacter radioresistens* S13 exposed to benzoic acid and phenol upregulated several proteins related to cell wall biogenesis, repair and the envelope stress response [58], indicating that aromatic organics stimulate the rearrangement of surface architecture and composition, consistent with this study.

Rhizobia produce an excessive amount of diverse surface polysaccharides such as lipopolysaccharides, gel-like, capsular, cyclic β-glucan, and neutral polysaccharides that are crucial for increased adhesion to abiotic surfaces and biofilm formation [59]. Continuous units of monosaccharides such as D-glucose, D-galactose, D-mannose, L-rhamnose, D-glucuronic acid and D-galacturonic acid, substituted with non-carbohydrate residues (e.g., acetyl, pyruvyl, succinyl and 3-hydroxybutanoyl groups) and their exposed—OH groups induce adhesion and alter surface physical properties [16,38,60,61]. Increased adhesion and elastic modulus in *Rlv* post 2,4-D treatment is a possible indication of increased biofilm formation due to 2,4-D adaptation, consistent with the extracellular material observed by SEM and AFM. Based on its lipophilic nature, 2,4-D is capable of entering the cell passively, altering membrane fluidity and permeability barriers as demonstrated for *Rhizobium sp*. M4 [33]. Thus, surface ultrastructural perturbations could also reflect underlying membrane perturbations as observed for similar aromatic organics with uncoupling properties [62–65].

### 2,4-D causes oxidative stress induced damage in *Rlv*

*Rlv* exposed to 2,4-D showed high levels of ROS (Fig 5 A), known to cause oxidative damage to envelope macromolecules, and a dose-dependent increase in carbonylated proteins (Fig 5 B) as a direct consequence of oxidative damage. Carbonyl (CO-) groups, produced upon oxidation of peptide side chains, are chemically stable and known to be more sensitive and direct indicators of oxidative stress [66]. The majority of aromatic and chlorinated aromatic hydrocarbons induce oxidative stress in bacteria [67–71], which has been attributed to their lipophility, reactivity and uncoupling capabilities. The decrease in pyrimidine metabolites such as thymidine, uridine 5-phosphate and uracil is indicative of DNA damage resulting from oxidative stress. Down-regulation of nucleotide metabolism has been observed for *E*. *coli* in response to cold, heat, oxidative stress and during its stationary phase [72]. Metabolite profiling indicated an increase in several polyamine metabolites such as cadaverine and putrescine involved in glutathione metabolism, that are known to serve as biomarkers of oxidative stress [73–75]. Increased polyamine biosynthesis has been associated with increased growth rate and increased protection from DNA damage due to oxidative stress [76]. Indeed, polyamine catabolism is initiated by the stationary phase stress transcription factor, σ^S^, producing a core metabolic stress response for a variety of environmental cues [74]. Therefore, we propose that increased levels of polyamines is an adaptive metabolic modification to cope with 2,4-D-induced oxidative stress.

### 2,4-D alters peptidoglycan and damages intracellular protein

The majority of significantly altered metabolites were positively correlated with 2,4-D treatment, including a large group of amino acids which may indicate proteolysis and protein denaturation [77] or a consequent upregulation of amino acid biosynthetic pathways to replace damaged proteins in the cell. Amino acid accumulation was observed in *E*. *coli* exposed to alcohols, heat, cold and oxidative stress, all likely due to protein denaturation and degradation [72]. A proteomic analysis of *Acinetobacter radioresistens* exposed to benzoate and phenol showed an upregulation in the majority of periplasmic proteases, chaperones, enzymes catalyzing peptidoglycan biogenesis, and proteins involved in outer membrane integrity, cell surface properties and cellular redox homeostasis [58], all consistent with this study. The reduction in peptidoglycan components along with the accumulation of alanine, lysine and glutamate implicates 2,4-D in cell wall damage, consistent with *C*. *glutamicum* which expresses cell wall biosynthetic enzymes [22] during 2,4-D exposure. Peptidoglycan damage is also consistent with the observed changes to envelope compliance and roughness in response to 2,4-D exposure.

### Adaptation to 2,4-D stress is an energy consuming process

Interestingly, *Rlv* grown in the presence of 2,4-D showed increased levels of glycolytic and TCA cycle metabolites, including citrate, phosphoenol pyruvate (PEP), glucose 6-phosphate (G-6-P), oxoglutarate and succinate, contrary to several studies of stressors at short exposure times. For example, reduced amounts of these intermediates were observed in *E*. *coli* during short time exposure to heat, cold and oxidative stress [72], whereas long term exposure experiments [78,79] are consistent with this study in which TCA cycle intermediates increased. *Burkholderia xenovorans* LB400 exposed to chlorobenzoate also showed induction of several TCA cycle enzymes [80]. In this study *Rlv* was grown to mid-log phase in the presence of 2,4-D, providing sufficient time to upregulate pathways that repair initial damage but require increased energy production. Increased levels of PEP and G-6-P also indicate a more active PEP-dependent glucose–phosphotransferase system (PTS), which acts as a center of carbohydrate flux in the cell and plays a significant role during nutrient starvation and stress adaptation in *E*. *coli* [81]. Since the PTS is actively involved in glucose transport and phosphorylation during glucose starvation, its upregulation indicates increased carbon use during 2,4-D adaptation. Upregulation of glycolysis, the TCA cycle and PTS intermediates signifies organized sensory, transport and energy production systems in *Rlv*, all representative of adaptive changes in response to 2,4-D-induced stress.

### *Rlv* has the potential to assimilate 2,4-D

An intriguing observation was the upregulation of the glyoxylate pathway, consistent with the 1.5 fold increase in malate synthase activity in 2,4-D treated *Rlv* (Fig 5 C). On the contrary, the activity of isocitrate lyase, the other key enzyme in the glyoxylate pathway, was unchanged with 2,4-D treatment (S4 Fig). These seemingly contradictory results could be explained if 2,4-D increases glyoxylate levels in the cell, thus specifically activating MS and not isocitrate lyase (ICL). Several 2,4-D degradation pathways have been discovered in soil bacteria and the intermediates of these pathways commonly enter the TCA cycle through glyoxylate [82–84]. 2,4-D degrading genes have been discovered in a closely related species, *Bradyrhizobium sp*. strain HW13 [85], however, to the best of our knowledge a 2,4-D degrading wild type *Rlv* strain has never been isolated. It is clear, however, that the glyoxylate cycle is upregulated during 2,4-D exposure to replenish the TCA cycle intermediates necessary for energy generation during 2,4-D adaptation. 2,4-D and related herbicides induce superoxide dismutase, malate dehydrogenase and auxin responsive genes in soybeans [86], consistent with *Rlv* exhibiting 2,4-D-induced oxidative stress, increased malate synthase activity and potential induction of auxin responsive genes, implied by the common phenotype in the presence of 2,4-D and auxin.

### Protein acetylation and myo-inositol, potential bioindicators of stress adaptation in *Rlv*

The 2,4-D treated *Rlv* had increased levels of acetyl lysine, indicative of a key post-translational modification related to intermediary metabolism [87]. A greater number of proteins are lysine-acetylated in the stationary phase, compared to those in the exponential phase [88], to regulate cell growth and proliferation [89]. It has been proposed that the majority of central metabolic enzymes are acetylated, and that this reversible process is an efficient means of adapting to changing environmental conditions [90]. Therefore, higher levels of acetyl lysine in the metabolite pool would be a key biomarker indicative of active adaptation to 2,4-D stress.

The increase in inositol phosphate metabolism in 2,4-D treated *Rlv*, as evidenced by the accumulation of myo-inositol, cannot be fully explained. The role of inositol phosphates in bacteria is not completely understood, but in eukaryotes, this pathway plays a significant role in signal transduction at the cell surface and regulation of membrane traffic, the cytoskeleton, and permeability and transport functions of membranes [91]. To date, accumulation of free myo-inositol has not been documented in prokaryotes [92], but this study indicates that it may play a significant role during stress adaptation in bacteria.

### 2,4-D upregulates membrane transport and ATP biosynthesis

Greater activity of the two component system, the ABC transport system, along with increased oxidative phosphorylation indicates a need to stimulate signal transduction and import vital macromolecules and ATP to fuel the observed adaptive changes, respectively, consistent with the upregulation of glycolysis and the TCA cycle. The membrane transport system plays a significant role during stress adaptation and this pathway is upregulated in *C*. *glutamicum* during 2,4-D stress [22] and in *E*. *coli* during nutrient limitation and oxidative stress [93]. Although ATP requirements are higher during active membrane transport and stress adaptation, several studies indicate a reduction in ATP biosynthesis [28,94], possibly resulting from reduced membrane integrity and the inability to adapt efficiently, especially during short term exposure. Indeed 2,4-D causes membrane defects, affects proton motive force and oxidative phosphorylation in *E*. *coli* [95] and *Comamonas testosteroni* [65]. Aromatic organic compounds in general, such as benzoate, chlorophenol and dinitrophenol, are known to accumulate in the cytoplasm, reduce internal pH, reduce growth and act as ‘uncouplers’, affecting membrane integrity, proton motive force and ATP biosynthesis [62–64].

Upregulation of ABC exporters is an adaptation mechanism that allows the export of xenobiotic substrates [96] and P-glycoproteins are known to interact with pesticides, playing a major role in toxicity resistance [97]. Increased PTS and membrane transport is required for the export of polysaccharide precursors for EPS production, an important adaptive response during stress resistance in bacteria [98] and consistent with the altered surface properties observed in *Rlv*. Upregulation of glycolysis, the TCA cycle, oxidative phosphorylation and the membrane transport system provides *Rlv* with the energy required for stress adaptation.

## Conclusions

Anthropogenic chemical compounds are known to disturb the legume-rhizobia symbiotic relationship, forcing rhizobia to reprogram integral pathways of cellular metabolism for adaptation with consequent changes to their cellular structures. This study shows that 2,4-D alters key pathways in *Rlv* central metabolism necessary for long term adaptation during 2,4-D exposure. Phenotypic changes in response to 2,4-D, similar to those found in bacteroids, can be ascribed to its structural relationship with auxin. Metabolites that responded specifically to 2,4-D exposure, namely acetyl-lysine, glyoxylate, myo-inositol and polyamines, can serve as sensitive biomarkers of stress adaptation in *Rlv*. This study provides valuable insights into the effects of 2,4-D exposure at sublethal levels on the beneficial model soil bacterium *R*. *leguminosarum*.

## Supporting Information

S1 Fig Representative SEM images of an altered phenotype for IAA treated cells. The majority of *Rlv* cells treated with 0.9 mM (A) and 0.4 mM (B) IAA showed an altered phenotype, with some cells appearing to be ‘Y-shaped’ and others branching or budding. *Rlv* treated with the benzoic acid negative control (C) were identical to controls. Scale bar A-C 1 μm
(TIF)

S2 Fig Representative AFM images of an altered phenotype for IAA treated cells. The majority of *Rlv* cells treated with 0.9 mM (a, A) and 0.4 mM (b, B) IAA showed an altered phenotype, with some cells appearing to be ‘Y-shaped’ and others branching or budding. This phenotype was similar to the bacteroids isolated from pea root nodules which also exhibited branching cells (c, C). *Rlv* treated with the benzoic acid negative control did not exhibit such a phenotype (d, D). Images a-d are low resolution (300 × 300) and A-D are high resolution (500 × 500). Scale bars, a-d = 1 μm, A-D = 0.5 μm.
(TIF)

S3 Fig Frequency of cells exhibiting an altered phenotype with each treatment. Cells treated with 0.9 mM IAA, 0.4 mM IAA and 0.4 mM 2,4-D had a significantly higher frequency of altered phenotypes compared to formula, control and benzoic acid treated samples (p < 0.0001, n ≥ 200).
(TIF)

S4 Fig ICL assay showing no difference in activity with 2,4-D treatment. Increase in phenylhydrazone derivative levels was measured as absorbance at 324 nm for 30 min. There was no difference in activity as a function of treatment type, as shown by the close parallel lines of all sample sets (p > 0.05).
(TIF)

S1 Methods Plant assay methods. (DOCX)

S1 Table Metabolomics data summary. Pathways altered in *Rlv* during 2,4-D exposure with associated metabolites, p-value, percentage and their KEGG IDs. For each annotation found in the set, MBRole reports a p-value which describes the probability of identifying a number of metabolites with a particular annotation compared to a random set of the same size within the background set. The percentage values shown for each pathway correspond to the percentage of altered metabolites associated with that pathway compared to the total number of altered metabolites. Metabolites from pathways in bold and regular font had higher and lower levels, respectively, compared to those of formula and control samples.
(DOCX)
