# Supplementary figures and images for: Rhizobium leguminosarum bv. viciae 3841 Adapts to 2,4-Dichlorophenoxyacetic Acid with “Auxin-Like” Morphological Changes, Cell Envelope Remodeling and Upregulation of Central Metabolic Pathways
Source: PLoS One. 2015 Apr 28;10(4):e0123813. doi: 10.1371/journal.pone.0123813 (PMC4412571; doi:10.1371/journal.pone.0123813)

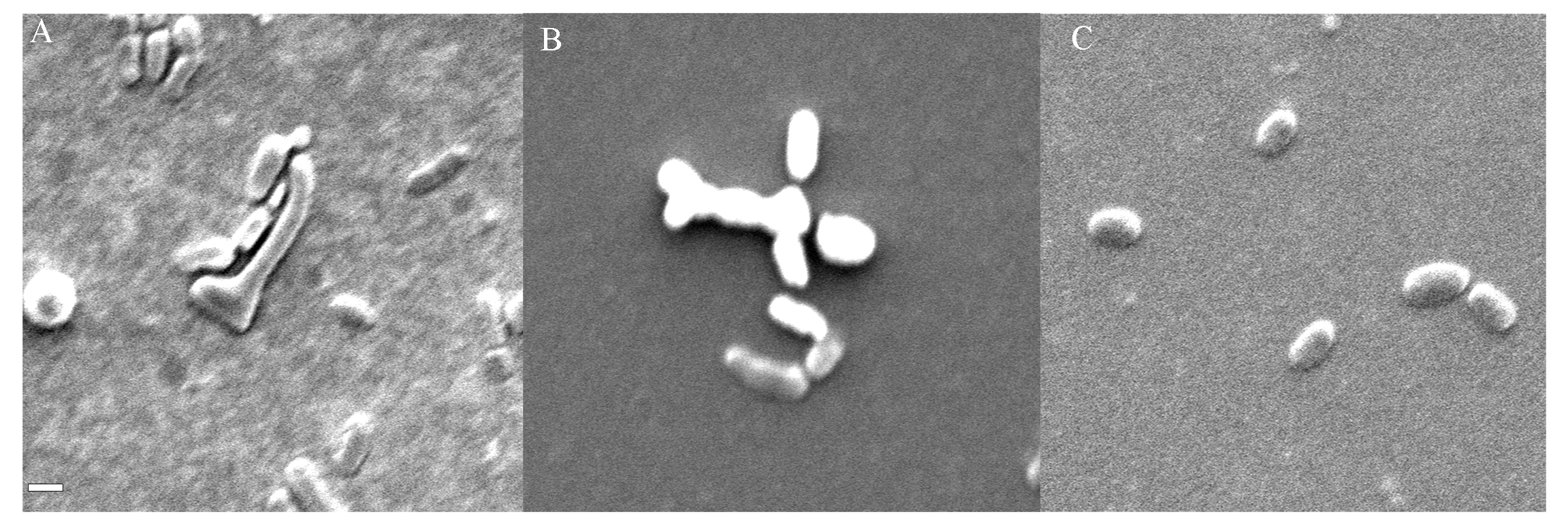

Supplement: S1 Fig — The majority of Rlv cells treated with 0.9 mM (A) and 0.4 mM (B) IAA showed an altered phenotype, with some cells appearing to be ‘Y-shaped’ and others branching or budding. Rlv treated with the benzoic acid negative control (C) were identical to controls. Scale bar A-C 1 μm (TIF) [file pone.0123813.s001.tif]

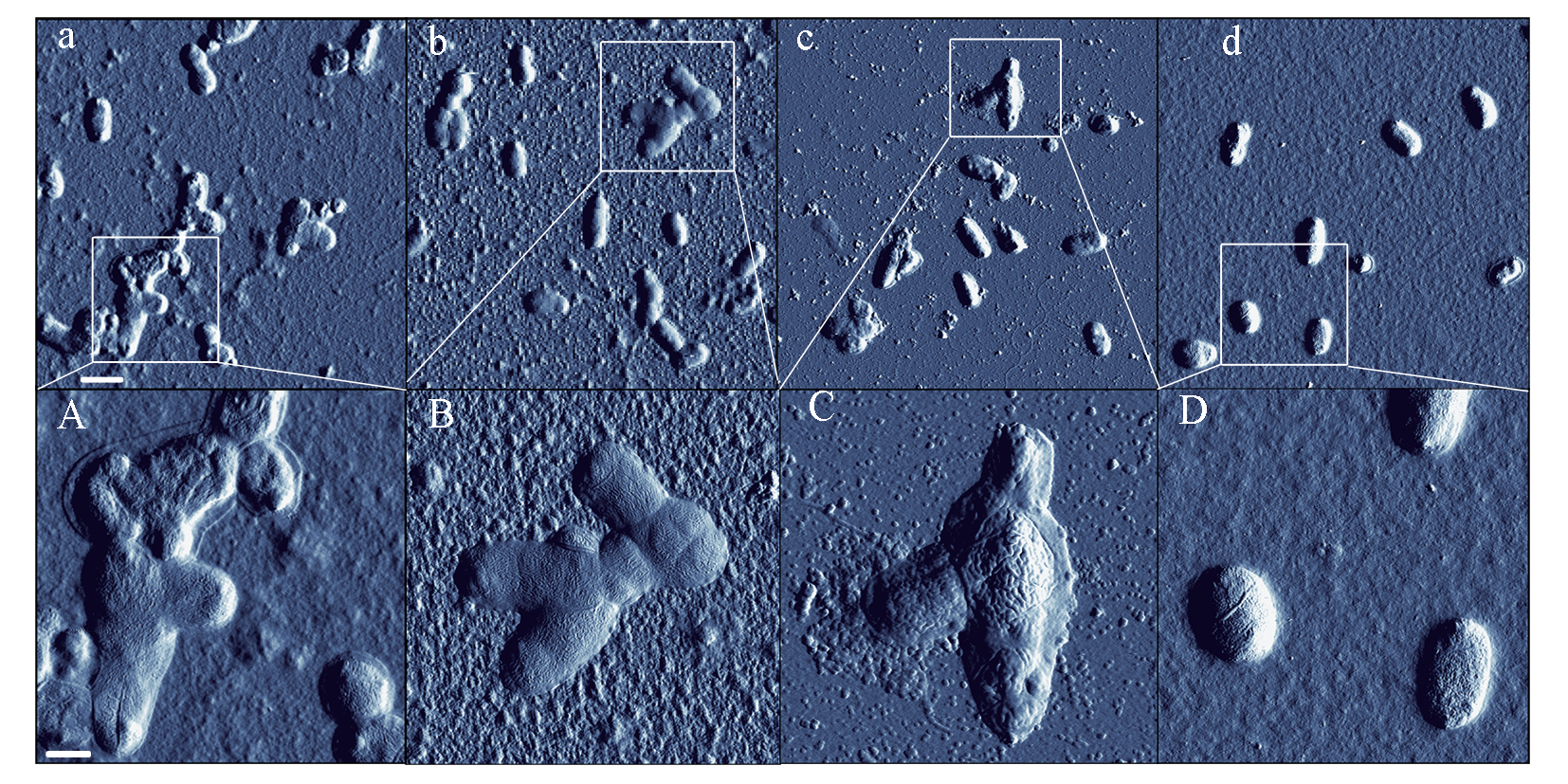

Supplement: S2 Fig — The majority of Rlv cells treated with 0.9 mM (a, A) and 0.4 mM (b, B) IAA showed an altered phenotype, with some cells appearing to be ‘Y-shaped’ and others branching or budding. This phenotype was similar to the bacteroids isolated from pea root nodules which also exhibited branching cells (c, C). Rlv treated with the benzoic acid negative control did not exhibit such a phenotype (d, D). Images a-d are low resolution (300 × 300) and A-D are high resolution (500 × 500). Scale bars, a-d = 1 μm, A-D = 0.5 μm. (TIF) [file pone.0123813.s002.tif]
