# Supplementary material for: Rhizobium leguminosarum bv. viciae 3841 Adapts to 2,4-Dichlorophenoxyacetic Acid with “Auxin-Like” Morphological Changes, Cell Envelope Remodeling and Upregulation of Central Metabolic Pathways
Source: PLoS One. 2015 Apr 28;10(4):e0123813. doi: 10.1371/journal.pone.0123813 (PMC4412571; doi:10.1371/journal.pone.0123813)
Supplement: S1 Methods — (DOCX) [file pone.0123813.s005.docx]

**Methods**

**Plant assays**

Nodulation assays were carried out with peas (*Pisum sativum* cv. Trapper) as the host legumes. Seeds were surface sterilized, germinated and planted as described previously [1]. Peas were inoculated with 500 µL of *Rlv* culture (O.D ~1) at the time of planting on vermiculite substrate. Nodules were harvested 5 weeks after planting.

**Microscopy**

To observe the morphology of bacteroids at high resolution they were imaged by AFM. Samples were prepared by crushing clean nodules onto PLL-coated coverslips between two clean, glass slides. After 5 min RT incubation, nodule debris was removed by a gentle rinse with buffer and coverslips were prepared as for the other samples (refer to Materials and methods).

**References**

1. Yost CK, Rochepeau P, Hynes MF. *Rhizobium leguminosarum* contains a group of genes that appear to code for methyl-accepting chemotaxis proteins. Microbiology. 1998; 144(Pt 7): 1945-1956.
