# Supplementary material for: Rhizobium leguminosarum bv. viciae 3841 Adapts to 2,4-Dichlorophenoxyacetic Acid with “Auxin-Like” Morphological Changes, Cell Envelope Remodeling and Upregulation of Central Metabolic Pathways
Source: PLoS One. 2015 Apr 28;10(4):e0123813. doi: 10.1371/journal.pone.0123813 (PMC4412571; doi:10.1371/journal.pone.0123813)
Supplement: S1 Table — Pathways altered in Rlv during 2,4-D exposure with associated metabolites, p-value, percentage and their KEGG IDs. For each annotation found in the set, MBRole reports a p-value which describes the probability of identifying a number of metabolites with a particular annotation compared to a random set of the same size within the background set. The percentage values shown for each pathway correspond to the percentage of altered metabolites associated with that pathway compared to the total number of altered metabolites. Metabolites from pathways in bold and regular font had higher and lower levels, respectively, compared to those of formula and control samples. (DOCX) [file pone.0123813.s006.docx]

| Pathway affected | p-value | Percentage | KEGG IDs | Metabolites |
| --- | --- | --- | --- | --- |
| Citrate cycle (TCA cycle) | 2.54E-05 | 12.1 | C00158 C00026 C00074 C00042 | Citric acid, oxoglutaric acid, phosphoenol pyruvate, succinate |
| Two-component system | 1.18E-04 | 12.1 | C00009 C00158 C00042 C00092 | Phosphoric acid, citric acid, succinic acid, |
| Glutathione metabolism | 3.45E-04 | 12.1 | C01672 C01879 C00037 C00134 | Cadaverine, pyroglutamic acid, glycine, putrescine |
| Oxidative phosphorylation | 3.61E-04 | 9.1 | C00009 C00042 C00013 | Phosphoric acid, succinic acid, pyrophosphate |
| Phosphotransferase system (PTS) | 7.25E-04 | 12.1 | C01083 C01452 C00074 C00092 | Trehalose, sorbose, phosphoenol pyruvate, glucose |
| ABC transporters | 0.001185 | 15.2 | C00037 C00134 C00093 C01083 C00009 | Glycine, putrescine, glycerol 3 phosphate, alpha-trehalose, orphophosphate |
| Glyoxylate and dicarboxylate metabolism | 0.007171 | 9.1 | C00026 C00158 C00042 | 2-oxoglutaric acid, citric acid, succinic acid |
| Lysine degradation | 0.008618 | 9.1 | C01672 C02727 C00037 | Cadaverine, acetyl-L-lysine, glycine |
| Pyrimidine metabolism | 0.016039 | 9.1 | C00106 C00105 C00214 | Uracil, uridylic acid, thymidine |
| Alanine, aspartate and glutamate metabolism | 0.01978 | 6.1 | C00026 C00042 | 2-oxoglutaric acid, succinate |
| Streptomycin biosynthesis | 0.01978 | 6.1 | C00137 C00092 | Myo-inositol, glucose-6-phosphate |
| Glycolysis / Gluconeogenesis | 0.023 | 6.1 | C00074 C00197 | Phosphoenolpuruvate, 3-phsophoglyceric acid |
| Pantothenate and CoA biosynthesis | 0.02473 | 6.1 | C00106 C00864 | Uracil, pantothenate |
| Peptidoglycan biosynthesis | 0.02473 | 6.1 | C00009 C00105 | Orthophosphate, uridine monophosphate, D-alanine |
| beta-Alanine metabolism | 0.032027 | 6.1 | C00106 C00864 | Uracil, pantothenate |
| Propanoate metabolism | 0.042181 | 6.1 | C00042 C02225 | Succinate, 2-methylcitric acid |
| Inositol phosphate metabolism | 0.04878 | 6.1 | C00092 C00137 | D-glucose-6-phosphate, myo-inositol |
| Arginine and proline metabolism | 0.024679 | 10.7 | C00134 C00064 C00334 | Putrescine, L-glutamine, 4-aminobutanoic acid |
| Galactose metabolism | 0.039 | 7.1 | C00137 C00243 | Myo-inositol, lactose |

**Table S1**: Metabolomics data summary.
